# Incidence and diagnostic yield of repeat imaging–based screening for hepatic arteriovenous malformations in patients with HHT

**DOI:** 10.1097/HC9.0000000000001010

**Published:** 2026-07-15

**Authors:** Benjamin Cho, Leena Usman, Torianna Lomax Truong, Darcy Phelan, Debajyoti Datta, Mandar A. Aras, Steven Hetts, Michael Ohliger, Miles Conrad, Jin Ge

**Affiliations:** 1 Division of Gastroenterology and Hepatology, Department of Medicine, University of California-San Francisco, San Francisco, California, USA; 2 Department of Radiology and Biomedical Imaging, Section of Abdominal Imaging, University of California-San Francisco, San Francisco, California, USA; 3 HHT Center of Excellence, University of California-San Francisco, San Francisco, California; 4 Division of Hematology and Oncology, Department of Medicine, University of California-San Francisco, San Francisco, California; 5 Division of Cardiology, University of California-San Francisco, Department of Medicine, San Francisco, California, USA; 6 Division of Neuroendovascular Surgery, Department of Radiology and Biomedical Imaging, University of California-San Francisco, San Francisco, California, USA; 7 Division of Interventional Radiology, University of California-San Francisco, Department of Radiology and Biomedical Imaging, San Francisco, California, USA

**Keywords:** diagnostic imaging, hereditary hemorrhagic telangiectasia (HHT), large language model (LLM), natural language processing (NLP), vascular malformations

## Abstract

**Background and Aims:**

Hepatic arteriovenous malformations (HAVMs) are a common visceral manifestation of hereditary hemorrhagic telangiectasia that can lead to heart failure and biliary ischemia. Current guidelines recommend baseline hepatic screening in adulthood, but the utility of repeat imaging after an initial negative or nonactionable finding remains unclear. We sought to clarify the prevalence of clinically significant HAVMs following negative or nonactionable imaging.

**Approach and Results:**

We isolated all adult patients with hereditary hemorrhagic telangiectasia evaluated at the University of California, San Francisco HHT Center of Excellence between 2013 and 2025. HAVM status was determined by natural language processing of unstructured clinical and radiology notes. Of 477 patients, 195 (40.9%) had initial imaging interpreted as either HAVM-negative or nonactionable, defined as HAVM-positive but not clinically significant to warrant further imaging. Of the 195, 30 (15.4%) underwent repeat hepatic imaging. Of the 30, 17 (56.7%) were screened incidentally and 13 (43.3%) were screened due to new signs and/or symptoms. Clinically significant HAVMs were identified in 2 patients, representing a 1.0% frequency of subsequent detection among all patients who were HAVM-negative/nonactionable and 6.7% among those re-imaged. Across 48 total repeat imaging studies, the detection rate per study was 4.2%. Across 386.5 person-years of follow-up, the incidence rate of clinically significant HAVM recognition was 0.52 per 100 person-years.

**Conclusions:**

Both instances of HAVM identification reflected evolving clinical features and reinterpretation of prior imaging, not de novo lesion development. These findings support periodic reassessment of initial negative/nonactionable screening studies, with a low threshold for reassessment in the presence of new signs and symptoms.

## Graphical abstract

**Figure:**
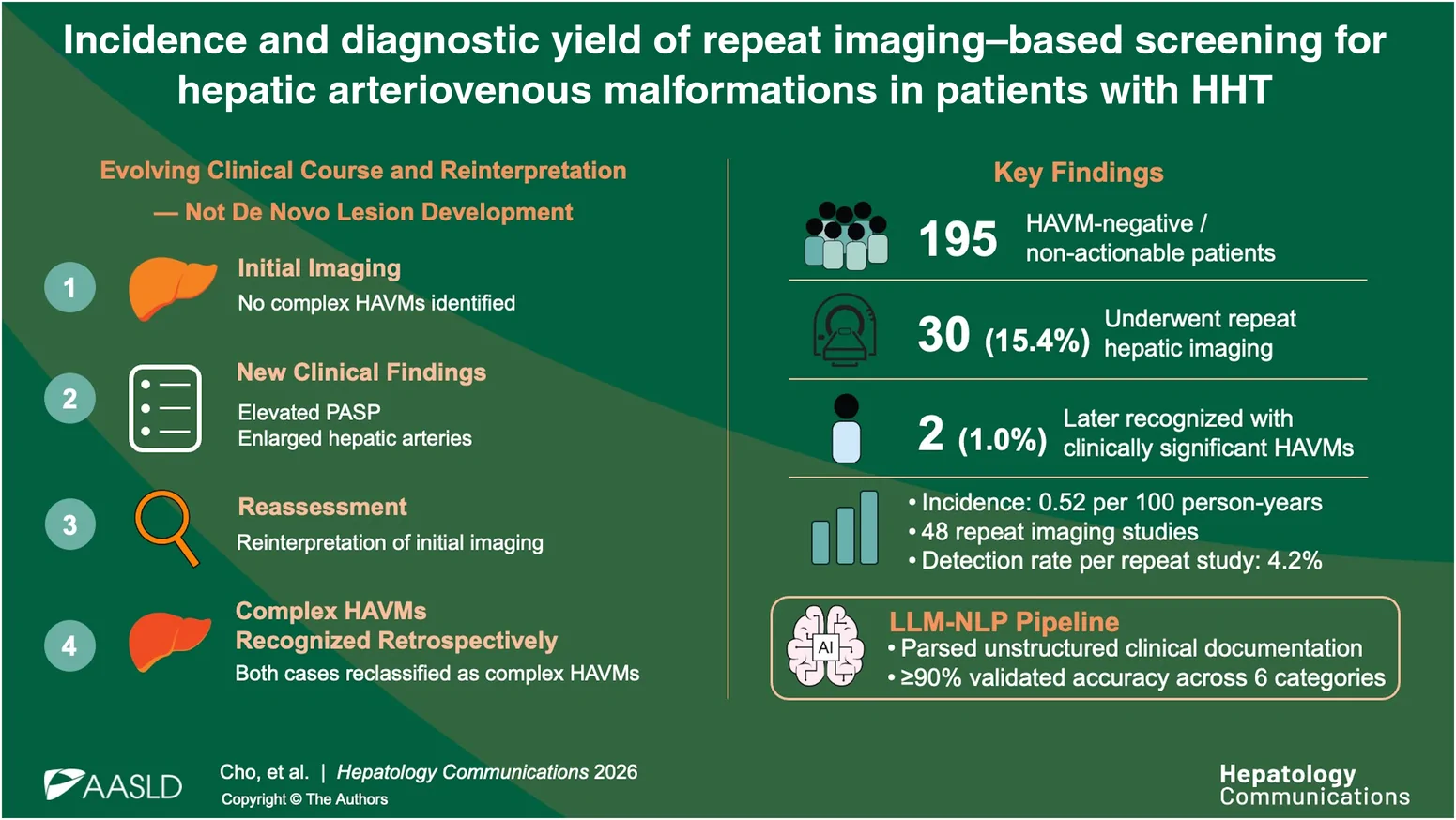

## INTRODUCTION

Hepatic arteriovenous malformations (HAVMs) are a well-recognized visceral manifestation of hereditary hemorrhagic telangiectasia (HHT), occurring in ~40%–70% of affected individuals when systematically screened.^1^^–^^3^ Although many remain asymptomatic, hepatic vascular shunting may eventually lead to high-output heart failure, portal hypertension, and/or biliary ischemia, making early detection and management clinically important.^3^^–^^5^

Current international HHT guidelines recommend baseline hepatic screening during adulthood at age 30, primarily with Doppler ultrasound, multiphase contrast CT, and MRI, to identify clinically significant HAVMs.^1^^,^^2^^,^^6^^–^^8^ Notably, the hemodynamic significance and imaging appearance of HAVMs may evolve over time, making interpretation complex and occasionally altering their perceived clinical relevance.^9^^–^^12^ As such, follow-up Doppler ultrasound and echocardiography is typically recommended for evaluation of disease progression.^1^^,^^13^

The vast majority of patients, however, either have a negative initial screen or have HAVMs that are deemed clinically insignificant or nonactionable.^2^^,^^7^^,^^10^^,^^14^ These patients are often recommended to have no additional screening tests or follow-up for their HAVMs unless there is the presence of new symptoms.^1^^,^^4^^,^^9^^,^^11^ Consequently, it remains unknown whether a single negative result is sufficient to exclude clinically relevant hepatic involvement or whether mandated periodic reassessment is needed to detect HAVM progression.

To address this gap, we evaluated the frequency and clinical context of subsequent clinically significant HAVMs among patients with HHT whose initial hepatic imaging was negative or nonactionable.

## METHODS

### Study design and patient selection

This study was approved by the UCSF Institutional Review Board under Study#17-22242, and all research was conducted in accordance with both the Declarations of Helsinki and Istanbul. We conducted a retrospective cohort study of adult (age ≥18 y) patients with HHT seen at the University of California, San Francisco (UCSF) HHT Center of Excellence (CoE) between January 1, 2013, and September 30, 2025. Patients were included in the cohort based on having (1) an International Classification of Diseases (ICD)-10 diagnosis code for HHT (I78.0), and (2) at least 1 documented HHT CoE clinic visit during the study period. Clinical encounters were queried from the UCSF deidentified clinical data warehouse to extract structured data and unstructured clinical notes. Patients (regardless of having an ICD-10 diagnosis code for HHT) without any notes associated with a HHT CoE encounter, or whose unstructured documentation explicitly indicated that HHT was “highly unlikely,” were excluded from the cohort.

### Data sources

Structured data were retrieved from the UCSF Clinical Data Warehouse, a Microsoft SQL Server–backed system accessed via Python (pyodbc) deployed within JupyterLab in a protected health information–compliant computing environment. For each patient, we extracted (1) unstructured progress notes linked to HHT CoE encounters, including the earliest available encounter and the most recent encounter within the study period; and (2) all abdominal imaging studies associated with HHT care, including radiology reports for abdominal ultrasound, CT, and MRI studies. Clinically relevant information about hepatic vascular malformations is predominantly documented in unstructured text, data collection and processing included complete radiology report impressions, provider assessments, and HHT-specific clinic notes. Therefore, all unstructured text was deidentified according to the Health Insurance Portability and Accountability Act standards before retrieval.

### Definition of repeat hepatic imaging

Repeat hepatic imaging was defined as any subsequent (incidental or for-cause) CT, MRI, or ultrasound of the abdomen or liver performed after an initial HAVM-negative or nonactionable study. Each repeat imaging study was categorized as (1) incidental, defined as performed for unrelated clinical reasons; or (2) HHT symptom-based, defined as performed due to new signs, symptoms, or complementary test abnormalities recognized in HHT CoE clinical visits. Categorization was performed by the same large language model (LLM)-natural language processing (NLP) pipeline as HAVM classification, achieving validated accuracy of 98.1% on a subset of 55 patients who had repeat imaging.

### LLM-assisted NLP for HAVM classification

Natural language processing was performed using a large-language-model–assisted pipeline built around the OpenAI-o1 model, accessed through the UCSF Versa API, a protected health information–compliant deployment of Microsoft Azure OpenAI at UCSF.^15^ We used the o1-2024-12-17 deployment running under the 2025-04-01-preview API version, and model reasoning effort was set to “medium.” For each patient, the OpenAI-o1 model reviewed the earliest HHT encounter note, the most recent HHT encounter note, and available abdominal imaging radiology reports. The primary NLP task was to classify each patient’s HAVM status as “HAVM-positive,” or “HAVM-negative/nonactionable.” The “nonactionable” category was defined as imaging findings positive for HAVMs but not complex or clinically significant enough to warrant further hepatic follow-up or imaging when reviewed during UCSF HHT CoE encounters. Noncomplex HAVMs were identified as vascular lesions associated with HHT that do not induce high flow states (eg, hemangiomas, focal nodal hyperplasia, and scattered perfusion abnormalities). This categorization was based on clinical consensus, with the main marker being nondilated hepatic arteries, indicating no increased flow. Secondary classification tasks included (1) identifying the pathogenic germline HHT variant of each patient; (2) determining whether further HAVM screening was recommended; (3) documenting the rationale for further screening or nonscreening; and (4) reporting the result of any hepatic imaging event.

We utilized zero-shot prompting, which provided the model with task-specific rules, definitions, and a required JSON schema without supplying any labeled examples. To prevent snowballing or cross-contamination of information across patients, each classification was executed as a standalone prompt with no retained conversational state. The model, therefore, processed each patient’s notes independently, using only the text supplied in that specific request. Prompts incorporated date metadata and explicit instructions on temporality, allowing the model to differentiate findings present at the first encounter from those that arose later. Each prompt specified a closed set of allowable labels (eg, “present,” “absent,” “missing”) and included rules to avoid misclassification due to family history, incidental findings, or unrelated organ systems. To ensure reproducibility, the prompt text required strict JSON-only output, which was validated by custom post-processing parsers. Full LLM prompts for each classification task are listed in Supplemental Materials 1, http://links.lww.com/HC9/C417.

LLM-NLP accuracy was validated against a manually abstracted 12% (66) randomly selected subset of patients. Trained clinical personnel blindly reviewed the same clinical progress notes and radiology reports that the OpenAI-o1 model was prompted on and independently classified each patient according to the same output structure as the model. Target LLM-NLP accuracy was defined as ≥ 90% validated accuracy in all categories before generalization.

### Clinician adjudication of LLM-derived classifications

Cases in which the LLM-NLP pipeline identified a change in HAVM status, defined as patients classified as HAVM-negative or nonactionable, for example, “no follow-up required,” at baseline who were later recognized as positive for complex HAVMs, then underwent independent clinical review. These charts were evaluated further by a group of multidisciplinary clinicians in the UCSF HHT CoE, who examined the original radiology reports, imaging studies when available, and associated clinical documentation to confirm LLM-derived HAVM recognition. This additional adjudication process served as the reference standard for determining the final classification of clinically progressive or evolving HAVMs and ensured that all clinically significant HAVM cases were accurately captured.

### Outcomes

The primary outcome was subsequent significant HAVM recognition in our cohort of patients who were HAVM-negative and nonactionable HAVM-positive. Secondary outcomes included per-round detection rate, defined as (newly recognized HAVMs)/(repeat imaging studies) × 100; and subsequent HAVM incidence rate, defined as (newly recognized HAVMs)/(total person-years of follow-up) × 100. CIs for incidence rates were estimated using nonparametric bootstrap resampling over 2000 repetitions.

## RESULTS

### Patient characteristics

A total of 542 patients with an ICD-10 diagnosis code for HHT and at least 1 documented clinical visit in the UCSF HHT CoE between January 1, 2013, and September 30, 2025, were screened for eligibility to our study cohort (Figure 1). We excluded 40 patients who were deemed to be “highly unlikely” to have HHT during a HHT CoE encounter, 25 patients who had no clinical notes attached to any HHT CoE encounters, and 282 patients who were classified as having complex clinically significant HAVMs or unknown HAVM status. As a result, there were 195 patients categorized as HAVM-negative/nonactionable, for example, not clinically significant or complex enough to warrant further hepatic follow-up, in the final analytical set. Patient characteristics, including demographics, HHT-related complications, and baseline laboratory studies closest to their initial HHT CoE encounter, are summarized in Table 1. Among patients with available genotypic data, ACVRL1 variants were most common, accounting for 37 of 43 (86.0%) identified genotypes.

**FIGURE 1:**
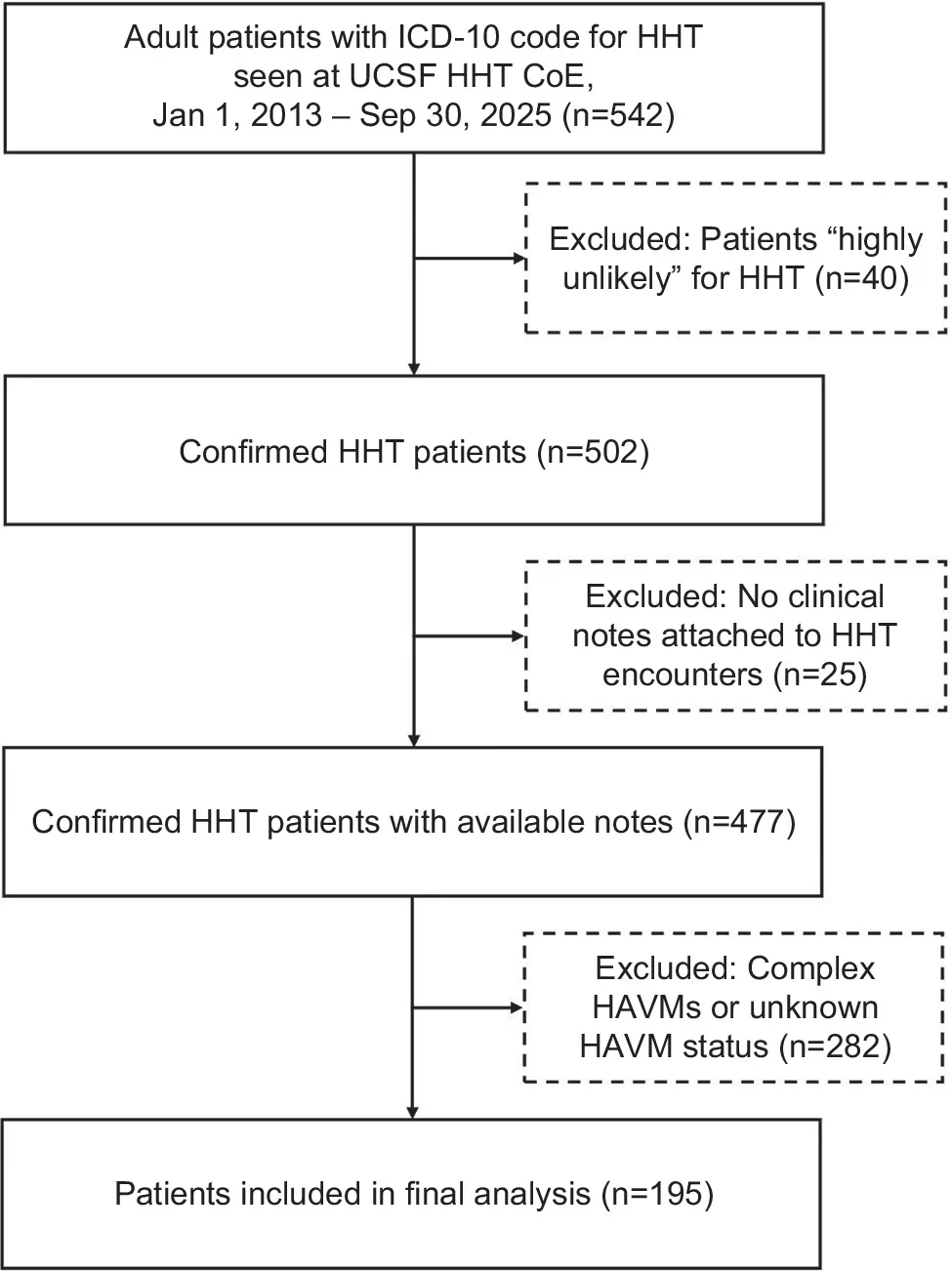
Patient selection. Abbreviations: CoE, Center of Excellence; HAVM, hepatic arteriovenous malformation; HHT, hereditary hemorrhagic telangiectasia; UCSF, University of California, San Francisco.

**TABLE 1:** Patient characteristics

Demographics	Overall (n=195)	
	Mean±SD	Range
Age (y)	45±17	18–82
Sex	121 female, 74 male	
Race		
White	116	
Asian	17	
Hispanic	37	
Black	3	
Other	8	
Unknown	14	
Genotype		
ACVRL1	37	
ENG	4	
SMAD4	1	
RASA1	1	
Negative testing	19	
Unknown	133	
AVM complications		
Brain AVMs	50	
Pulmonary AVMs	96	
GI bleeding	31	
Epistaxis severity score	3.24±2.36	0–9.04
Baseline labs		
WBC (10^3^/μL)	6.4±2.2	1.9–11.5
Hemoglobin (g/dL)	12.9±2.4	5.8–18.2
Hematocrit (%)	39.7±6.2	23.5–52.9
Platelet count (10^3^/μL)	253.7±71.3	108–448
AST (U/L)	28.1±26.3	11–190
ALT (U/L)	31.2±36.2	7–248
Alkaline phosphatase (U/L)	72.3±30.2	28–166
Total bilirubin (mg/dL)	0.7±0.4	0.1–1.9
Albumin (g/dL)	3.7±0.7	2.0–5.2
INR	1.1±0.2	0.9–2.2

Abbreviations: AVM, arteriovenous malformation; INR, International Normalized Ratio; WBC, White Blood Cell.

### LLM-NLP performance and validation

The LLM-NLP pipeline utilizing the OpenAI-o1 model demonstrated high accuracy (≥90%) across all major classification tasks (Table 2). In a manually reviewed subset of 66 patients, the model correctly identified baseline HAVM status in 60 (90.9%) and latest HAVM status in 62 (93.9%). The genotypic variant of HHT for each patient was abstracted correctly in 65 patients (98.5%). Screening recommendation classification was correctly identified in all 66 patients (100%), and screening rationale extraction was accurate in 54 of 55 patients with available rationale (98.2%). Lastly, HAVM findings extracted from radiology reports of hepatic imaging were successfully classified in 15 of 16 manually reviewed events (93.8%).

**TABLE 2:** LLM-NLP pipeline components and validation performance across classification tasks

Category	Description	N (validation subset)	Accuracy (%)
Baseline HAVM status	Presence/absence/unknown status of HAVMs at the earliest HHT clinic encounter	66	90.9% (60/66)
Latest HAVM status	Presence/absence/unknown status of HAVMs at the latest HHT clinic encounter	66	93.9% (62/66)
Genotype	Identification of pathogenic germline HHT variant	66	98.5% (65/66)
Hepatic imaging results	HAVM findings from radiology reports of hepatic imaging events	16	93.8% (15/16)
Screening recommendation	Yes/no/conditional/unknown clinical recommendation for HAVM screening	66	100% (66/66)
Screening rationale	Categorization of rationale for screening/nonscreening (symptoms, history of HAVMs, prior imaging results, etc)	55	98% (54/55)

Abbreviations: HAVM, hepatic arteriovenous malformation; HHT, hereditary hemorrhagic telangiectasia; LLM, large language model; NLP, natural language processing.

### Patients with repeat hepatic imaging

Of the 195 patients, 30 (15.4%) underwent repeat hepatic imaging, defined as any subsequent CT, MRI, or ultrasound of the abdomen or liver following a baseline imaging study. Of these, 17 (56.7%) were screened incidentally, that is, for clinical reasons unrelated to liver complications. Thirteen (43.3%) were screened due to new signs, symptoms, or complementary test abnormalities linked to HHT-related liver involvement recognized in HHT CoE clinical visits. Of 48 total repeat hepatic imaging events, 8 (16.7%) were CT, 15 (31.3%) were MRI, and 25 (52.1%) were Doppler ultrasound. The median time interval from initial HHT CoE encounter to first repeat imaging event was 2 months (range, 0–107). This relatively short interval likely reflects the inclusion of early follow-up studies performed to clarify indeterminate or incomplete initial imaging.

### Incidence of new clinically significant HAVMs and per-round detection rates

Two patients were found to have new clinically significant HAVMs in repeat hepatic imaging studies, representing a 1.0% frequency of evolving detection among all HAVM-negative/nonactionable patients and 6.7% among re-imaged patients. Across our cohort of 195 HAVM-negative/nonactionable patients, there were 386.5 total person-years of HHT CoE follow-up. The incidence rate of clinically significant HAVM recognition was thus estimated at 0.52 per 100 person-years (95% CI, 0.00–1.37). One patient had repeat screening due to new or evolving symptoms consistent with clinically significant HAVM (“HHT-symptom”), and the other had incidental imaging for another clinical indication.

In the entire cohort, there were 48 total imaging events, representing a per-round detection rate of 4.2% across all repeat hepatic imaging. Among the subset of 13 patients with HHT-symptom–based screening, there were 19 total imaging events, representing a per-round detection rate of 5.3%. There were 29 imaging studies in the 17 patients with incidental screening, equating to a per-round detection rate of 3.4%.

### Evolving clinical courses in new clinically significant HAVM recognition

Both instances of later significant HAVM identification reflected an evolving clinical course, prompting reinterpretation of prior imaging, rather than the true biological development of new HAVMs. In one patient, an initial abdominal MRI at age 61 prompted a recommendation of no further hepatic workup, with findings of hyperenhancing lesions suggestive of focal nodal hyperplasia. Approximately 2 years later, new signs and symptoms, including elevated pulmonary artery systolic pressure on transthoracic echocardiography attributable to a clinically significant HAVM, prompted reassessment of prior imaging.

In the second patient, initial evaluation of an initial CT abdomen/pelvis at age 61 similarly concluded that no further hepatic imaging was required. Repeat imaging occurred incidentally ~5 years later, during a CT pulmonary embolism performed to evaluate for pulmonary AVMs. This chest study raised suspicion for HAVMs due to enlarged and tortuous hepatic arteries in the captured abdominal images, prompting retrospective reassessment of the original abdominal CT. The same findings were subsequently recognized, and clinical recommendations were revised to reflect management of clinically significant HAVMs.

## DISCUSSION

This study demonstrates that the development of clinically significant HAVMs in patients with HHT with initial negative or nonactionable hepatic imaging is rare but not negligible. Among 195 patients with baseline evaluations prompting no further follow-up, 2 were later identified as having clinically significant HAVMs. More importantly, in both cases, HAVM recognition reflected an evolving clinical course and reinterpretation of prior imaging rather than true biological development of new lesions. While both patients were ultimately re-categorized to the complex HAVM group based on initial imaging findings, given that initial clinical management was in the HAVM-negative/nonactionable group, they still belong to the negative/nonactionable cohort based on the clinical decision-making at the time of initial categorization. To our knowledge, this represents the largest retrospective cohort study of patients with HHT investigating clinically significant HAVM prevalence among patients with negative or nonactionable baseline hepatic screening.

Our results suggest that initially HAVM-negative/nonactionable patients who demonstrate signs of clinical evolution, such as elevated liver function tests, abdominal pain, or signs of heart failure, may warrant a particularly low threshold for re-evaluation.^5^^,^^11^ At the UCSF CoE, a standardized imaging protocol ensures all patients without complex HAVMs on prior imaging undergo Doppler ultrasound for grading after their initial visit, with further evaluation by phase-contrast MRI with flow measurements in addition to monthly multidisciplinary review when complex features are identified. Notably, in both patients with subsequent significant HAVM recognition, new clinical information, including elevated pulmonary artery systolic pressure and enlarged hepatic arteries in incidental CT imaging, prompted reassessment of prior imaging, ultimately leading to rescreening and confirmation of the presence of clinically significant HAVMs. Current international guidelines strongly recommend initial diagnostic screening in patients with symptoms linked to complicated HAVMs, suggesting that reinterpretation of prior imaging and/or repeat diagnostic imaging should be considered for initially nonactionable patients who develop such signs and symptoms.^1^ In addition, both patients with subsequent HAVM recognition had imaging initially interpreted as HAVM-negative later reclassified as HAVM-positive, reflecting the potential for clinical misinterpretation and reassessment following an evolving clinical course. The observed HAVM incidence rate of 0.52 per 100 person-years demonstrates the relevance of periodic clinical reinterpretation to identify evolving or previously under-recognized HAVMs, rather than an explicit need for de novo disease surveillance.

This study demonstrates the feasibility and reliability of LLM–assisted NLP for extracting structured variables from complex, unstructured clinical documentation. Accuracy rates across all categories utilizing the OpenAI-o1 LLM exceeded ≥90%, supporting the validity of LLM-assisted classification within this retrospective cohort. The LLM performed notably well on identifying the genotypic variant of HHT and whether hepatic screening was recommended for each patient with accuracy rates of 98.5% (65/66) and 100% (66/66), respectively. This performance suggests greater NLP reliability in variables generally stated explicitly in unstructured text with little need for model inference. The high level of NLP accuracy in all 6 categorization tasks demonstrates the usefulness of NLP in efficient, systematic data extraction from large sets of unstructured documentation. Future studies may find value in utilizing such an LLM-NLP pipeline to streamline cohort identification in a similar manner to this study. Our results suggest NLP viability in nonstudy use cases as well, such as in surveillance programs or general cohort categorization. Future applications of AI in HHT care may also include stratifying HAVM complexity and identifying patients who warrant closer follow-up or repeat imaging based on evolving clinical features. Nonetheless, manual clinician review of LLM results remains essential for validating model outputs and ensuring that final classifications reflect true clinical interpretations. Specialist chart review and evaluation at an HHT CoE remains the gold standard in identifying changes in HAVM status.

Lastly, the economic and clinical tradeoffs of repeat hepatic imaging in patients with HAVM-negative/nonactionable HHT remain undefined.^9^ Although Doppler ultrasound is considered an effective, low-cost study for initial screening, it is unknown whether routine repeated interval-based screening of any modality is beneficial enough to justify increased patient costs and safety risks.^1^^,^^2^^,^^6^^,^^16^ Prospective studies incorporating cost-effectiveness analyses, quality-of-life measures, and standardized surveillance protocols are needed to determine whether targeted or scheduled rescreening strategies are warranted or can be optimized.

We acknowledge several limitations in our study. First, this was a retrospective, single-center analysis, which may limit generalizability to other practice settings with different patient populations, imaging practices, or thresholds for defining clinically significant HAVMs. Second, repeat hepatic imaging was not performed according to a standardized surveillance protocol but occurred incidentally or in response to evolving clinical features. Patients with repeat hepatic imaging had a median of 4 subsequent visits to the UCSF HHT CoE (range, 0–19), whereas patients who were never re-imaged had a median of 1 subsequent visit (range, 0–21); this higher rate of surveillance for re-imaged patients introduced possible selection bias to the observed HAVM detection and incidence rates. Third, HAVM classification relied on unstructured clinic notes and radiology impressions, and although the LLM-NLP pipeline was validated to achieve ≥90% accuracy across all categories and all cases of HAVM recognition underwent clinician review, misclassification remains possible, particularly in cases with ambiguous documentation. Finally, because clinical text was programmatically deidentified to meet protected health information compliance requirements, some information relevant to secondary analyses was removed. This most notably affected HHT genotypic variant classification; although overall LLM-NLP genotype classification accuracy was high (98.5%), the majority of patients’ specific genotypic variants were unavailable.

## CONCLUSIONS

In this retrospective cohort of patients with HHT with initially negative or nonactionable hepatic imaging, subsequent recognition of clinically significant HAVMs was uncommon but not negligible. In the 2 patients where significant HAVMs were later identified, they reflected evolving clinical features and reinterpretation of prior imaging rather than the clear emergence of new lesions. These findings suggest that while routine interval-based hepatic rescreening may not be warranted for all patients, initial negative or nonactionable imaging should be periodically revisited, with a particularly low threshold for reassessment in patients where new signs, symptoms, or complementary imaging findings emerge.

## Supplementary Material
