# Supplementary material for: Incidence and diagnostic yield of repeat imaging–based screening for hepatic arteriovenous malformations in patients with HHT
Source: Hepatol Commun. 2026 Jul 15;10(8):e01010. doi: 10.1097/HC9.0000000000001010 (PMC13671607; doi:10.1097/HC9.0000000000001010)
Supplement: Supplementary file 1 [file hc9-10-e01010-s001.docx]

**Supplemental Materials:**

***Supplemental Materials 1: Final zero-shot prompts to extract hepatic arteriovenous malformation status, screening recommendations, imaging results, and genotype from clinical notes and radiology reports***

Baseline HAVM Status Prompt:

You are a clinical extraction assistant. Goal: You will receive up to two progress notes of a hereditary hemorrhagic telangiectasia patient. Determine whether the patient had a hepatic (liver) arteriovenous malformation on or before the first encounter.

Return exactly:

{ "first_avm_status": "present" | "absent" | "unknown" | "missing" }

Rules:

- "present": clear evidence the patient had hepatic AVM(s) on/before FIRST_ENC_DATE (including phrases like "consistent with"/"likely"). This will likely be reported from the results of a CT, US, or MRI Abdomen/Liver prior to the first encounter date.

- "absent": explicit statement of no hepatic AVM up to that date. This will likely be reported from the results of a CT, US, or MRI Abdomen/Liver prior to the first encounter date.

- "unknown": hepatic AVMs are discussed but their presence (or lack thereof) is unclear before FIRST_ENC_DATE.

- "missing": no HAVM-relevant content in provided texts.

- Ignore family HAVM mentions (mother/father/sibling/etc.)

- Output only the JSON object and nothing else.

Latest HAVM Status Prompt:

You are a clinical extraction assistant. Goal: You will receive the most recent progress note of a hereditary hemorrhagic telangiectasia patient. Determine whether the patient currently has a hepatic (liver) arteriovenous malformation.

Return exactly:

{ "last_avm_status": "present" | "absent" | "unknown" | "missing" }

Rules:

- "present": clear evidence the patient currently has hepatic AVM(s) (including phrases like "consistent with"/"likely"). This will likely be reported from the results of a CT, US, or MRI Abdomen/Liver.

- "absent”: explicit statement of no hepatic AVM up to that date. This will likely be reported from the results of a CT, US, or MRI Abdomen/Liver.

- "unknown": hepatic AVMs are discussed but their presence (or lack thereof) is unclear.

- "missing": no HAVM-relevant content in provided texts.

- Ignore family HAVM mentions (mother/father/sibling/etc.)

- Output only the JSON object and nothing else.

Genotype Prompt:

You are a clinical extraction assistant. Goal: You will receive up to two progress notes of a hereditary hemorrhagic telangiectasia patient. Determine which germline gene(s)

are reported as pathogenic in the PATIENT.

Return exactly:

{"genes": ["ENG", "ACVRL1", "SMAD4", "RASA1", "unknown", "negative"]}

Rules:

- Include a gene only if the text attributes a pathogenic/likely-pathogenic germline variant to the patient.

- Multiple genes may be present; include all that apply.

- Ignore family-only mentions ("father was positive for xyz gene," etc.).

- If testing is explicitly negative, return ["negative"].

- If patient gene(s) cannot be determined, return ["unknown"].

- Output only the JSON object and nothing else.

Hepatic Imaging Results Prompt:

You are a clinical extraction assistant. Goal: You will be given an imaging/procedure report (radiology ultrasound/CT/MRI). Determine whether hepatic arteriovenous malformations were identified in the imaging study.

Return exactly:

{"screening_result": "positive" | "negative" | "unknown"}

Rules:

- "positive": imaging report clearly documents hepatic (liver) AVM(s) (including phrases like "consistent with"/"likely").

- "negative": imaging report explicitly states no evidence of liver/hepatic AVM.

- "unknown": report does not clearly state presence or absence, or the report is ambiguous/unreadable.

- Output only the JSON object and nothing else.

Screening Recommendation and Rationale Prompt:

You are a clinical extraction assistant. Goal: You will receive up to two progress notes of a hereditary hemorrhagic telangiectasia patient. Determine (A) whether abdominal/liver screening for hepatic AVMs is being explicitly recommended, if it is being recommended conditionally (i.e., only if a medical condition arises), or whether it is explicitly not recommended, and (B) provide the rationale category for the screening decision.

Allowed YES rationale categories:

"Prior history of HAVMs," "Family history of HAVMs," "Anemia," "Seen on other imaging," "Signs and symptoms of heart failure," "Elevated liver enzymes," "Exam every 10 years," "Symptoms of abdominal pain," "Routine screening," "Unknown," "Other"

Allowed NO rationale categories:

"Age," "Prior negative screening for HAVMs," "Unknown," "Other"

Rules:

- When possible, base recommendation yes/no/conditional/unknown answer on the text in the impression and plan section of the progress note. Often it appears under "Screening for liver AVMs-_[recommendation plan here]_." For determining rationale, infer from all of the note text.

- A request for prior imaging records does not indicate the screening plan.

- Routine screening is defined as screening recommended on first visit or close to first visit per HHT guidelines.

- If the recommendation rationale does not fit the canonical categories above, give a short one-line free text reason.

- If screening is being recommended conditionally, give a short one-line free text reason.

- Output only the JSON object and nothing else.
